# 1-Methyl-4-[(*E*)-2-(2-thien­yl)­ethen­yl]­pyridinium 4-methyl­benzene­sulfonate^1^

**DOI:** 10.1107/S1600536808031401

**Published:** 2008-10-04

**Authors:** Suchada Chantrapromma, Pumsak Ruanwas, Hoong-Kun Fun, Chatchanok Karalai

**Affiliations:** a Crystal Materials Research Unit, Department of Chemistry, Faculty of Science, Prince of Songkla University, Hat-Yai, Songkhla 90112, Thailand; b X-ray Crystallography Unit, School of Physics, Universiti Sains Malaysia, 11800 USM, Penang, Malaysia

## Abstract

In the title compound, C_12_H_12_NS^+^·C_7_H_7_O_3_S^−^, the cation exists in an *E* configuration with respect to the ethenyl C=C bond. The cation is essentially planar with a dihedral angle of 1.94 (10)° between the pyridinium and thio­phene rings. The benzene ring of the anion makes dihedral angles of 75.23 (10) and 76.83 (10)°, respectively, with the pyridinium and thio­phene rings. In the crystal structure, cations and anions form alternate layers parallel to the *bc* plane. Within each layer, both cations and anions are arranged into chains directed along the *b* axis. The cation chain and the anion chain are inter­connected by weak C—H⋯O inter­actions into a three-dimensional network. The crystal structure is further stabilized by C—H⋯π inter­actions.

## Related literature

For bond lengths, see: Allen *et al.* (1987 ▶). For related literature on hydrogen-bond motifs, see: Bernstein *et al.* (1995 ▶). For related structures, see, for example: Chantrapromma, Jindawong & Fun (2007 ▶); Chantrapromma, Jindawong, Fun & Patil (2007 ▶); Chantrapromma *et al.* (2008 ▶); Lakshmanaperumal *et al.* (2002 ▶, 2004 ▶); Rahman *et al.* (2003 ▶); Ruanwas *et al.* (2008 ▶); Usman *et al.* (2000 ▶, 2001 ▶).
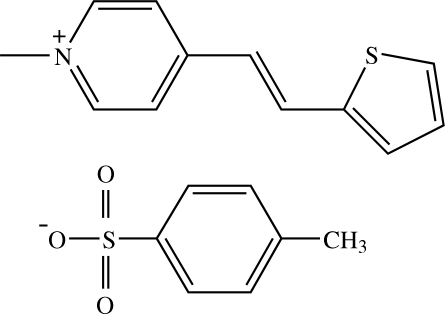


## Experimental

### 

#### Crystal data

C_12_H_12_NS^+^·C_7_H_7_O_3_S^−^
*M* *_r_* = 373.49
Triclinic, 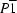

*a* = 9.2947 (1) Å
*b* = 9.6144 (1) Å
*c* = 10.7790 (1) Å
α = 87.817 (1)°
β = 64.702 (1)°
γ = 88.712 (1)°
*V* = 870.21 (1) Å^3^
*Z* = 2
Mo *K*α radiation
μ = 0.32 mm^−1^
*T* = 100.0 (1) K
0.36 × 0.35 × 0.18 mm

#### Data collection

Bruker SMART APEXII CCD area-detector diffractometer
Absorption correction: multi-scan (**SADABS**; Bruker, 2005 ▶) *T*_min_ = 0.893, *T*_max_ = 0.945
18024 measured reflections
4606 independent reflections
4122 reflections with *I* > 2σ(*I*)
*R*_int_ = 0.023

#### Refinement

*R*[*F* ^2^ > 2σ(*F* ^2^)] = 0.051
*wR*(*F* ^2^) = 0.148
*S* = 1.04
4606 reflections
228 parameters
H-atom parameters constrained
Δρ_max_ = 0.98 e Å^−3^
Δρ_min_ = −0.71 e Å^−3^

### 

Data collection: *APEX2* (Bruker, 2005 ▶); cell refinement: *SAINT* (Bruker, 2005 ▶); data reduction: *SAINT*; program(s) used to solve structure: *SHELXTL* (Sheldrick, 2008 ▶); program(s) used to refine structure: *SHELXTL*; molecular graphics: *SHELXTL*; software used to prepare material for publication: *SHELXTL* and *PLATON* (Spek, 2003 ▶).

## Supplementary Material

Crystal structure: contains datablocks global, I. DOI: 10.1107/S1600536808031401/is2338sup1.cif

Structure factors: contains datablocks I. DOI: 10.1107/S1600536808031401/is2338Isup2.hkl

Additional supplementary materials:  crystallographic information; 3D view; checkCIF report

## Figures and Tables

**Table 1:** Hydrogen-bond geometry (Å, °)

*D*—H⋯*A*	*D*—H	H⋯*A*	*D*⋯*A*	*D*—H⋯*A*
C2—H2*A*⋯O3^i^	0.93	2.31	3.219 (3)	166
C3—H3*A*⋯O1^ii^	0.93	2.49	3.168 (2)	130
C6—H6*A*⋯O2	0.93	2.56	3.378 (3)	147
C11—H11*A*⋯O1^iii^	0.93	2.54	3.303 (3)	139
C12—H12*A*⋯O1^i^	0.96	2.52	3.455 (3)	165
C12—H12*C*⋯O1^ii^	0.96	2.47	3.341 (3)	151
C15—H15*A*⋯O2^iv^	0.93	2.42	3.272 (2)	152
C17—H17*A*⋯O3^i^	0.93	2.43	3.202 (2)	141
C4—H4*A*⋯*Cg*1^v^	0.93	2.62	3.431 (2)	145
C10—H10*A*⋯*Cg*1^vi^	0.93	2.95	3.666 (3)	135

Symmetry codes: (i) 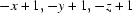; (ii) 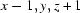; (iii) 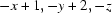; (iv) 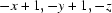; (v) 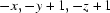; (vi) 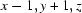. *Cg*1 is the centroid of the C13–C18 benzene ring.

**Fig. 1.**
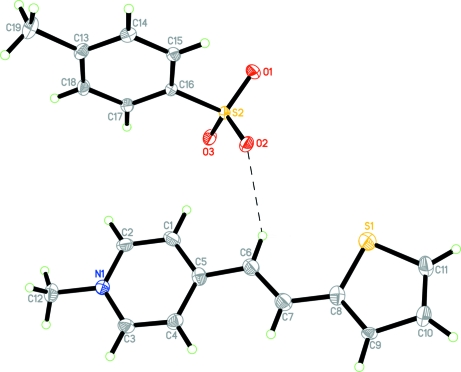
The molecular structure of the title compound, showing 50% probability displacement ellipsoids and the atom-numbering scheme.

**Fig. 2.**
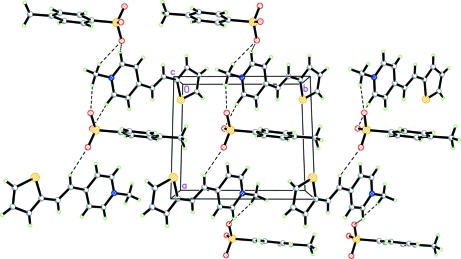
The packing diagram of the title compound, viewed along the c axis. The weak C—H···O and C—H···S interactions are drawn as dashed lines.

## supplementary crystallographic information

### Comment

Pyridinium derivatives have been found to have nonlinear optical properties (Lakshmanaperumal *et al.*, 2002, 2004; Usman *et al.*, 2000, 2001). We have previously synthesized and crystallized several compounds of pyridinium and quinolinium derivatives to study their non-linear optical properties (Chantrapromma, Jindawong & Fun, 2007; Chantrapromma, Jindawong, Fun & Patil, 2007; Chantrapromma *et al.*, 2008; Ruanwas *et al.*, 2008). As part of our research on nonlinear optic materials, the title compound was synthesized.

The asymmetric unit of the title compound consists of the C_12_H_12_NS^+^ cation and the C_7_H_7_O_3_S^-^ anion. The cation exists in an *E* configuration with respect to the ethenyl C═C bond [C6═C7 = 1.346 (3) Å]. The cation is essentially planar with a dihedral angle between the pyridinium and thiophene rings of 1.94 (10)°. The orientation of the anion with respect to the cation can be indicated by the interplanar angles between the benzene ring [C13–C18] with the pyridinium [C1–C5/N1] and thiophene [C8—C11/S1] rings of 75.23 (10) and 76.83 (10)°, respectively. The ethenyl unit is nearly coplanar with the pyridinium and thiophene rings with the torsion angles C4–C5–C6–C7 = 3.0 (3)° and C6–C7–C8–S1 = -3.7 (3)°. The atom O3 of the sulfonate and the S1 atom of the thiophene contribute to the weak intramolecular C—H···O and C—H···S interactions, forming S(5) ring motifs (Bernstein *et al.*, 1995). The bond lengths and angles are normal (Allen *et al.*, 1987) and are comparable with closely related structures (Chantrapromma, Jindawong & Fun, 2007; Chantrapromma, Jindawong, Fun & Patil, 2007; Chantrapromma *et al.*, 2008; Ruanwas *et al.*, 2008).

All the O atoms of 4-methylbenzenesulfonate anion are involved in the C—H···O weak interactions (Table 1). In the crystal packing (Fig. 2), the cations and anions form alternate layers parallel to the *bc* plane. Within each layer both cations and anions are arranged into chains directed along the *b* axis. The cations and anions chains are interconnected by C—H···O weak interactions into a three dimensional network. The crystal structure is further stabilized by the C4—H4A···π and C10—H10A···π interactions (Table 1); *Cg*_1_ is the centroid of the C13–C18 benzene ring.

### Experimental

The title compound was synthesized by mixing 4-(2-thiophenestyryl)-1-methylpyridinium iodide (0.1 g, 0.3 mmol) which was prepared in a similar manner to that previously reported (Chantrapromma *et al.*, 2008) in hot methanol (40 ml) and *p*-toluenesulfonate (0.09 g, 0.3 mmol) in hot methanol (30 ml) (Rahman *et al.*, 2003). The mixture immediately yielded a yellow solid of silver iodide. After stirring the mixture for 30 min, the precipitate of silver iodide was removed and the resulting solution was evaporated and the green-yellow solid was obtained. Yellow block-shaped single crystals of the title compound suitable for *x*-ray structure determination were recrystalized from the methanol/ethanol (1:1 *v*/*v*) solvent by slow evaporation of the solvent at room temperature after several weeks (m.p. 507–509 K).

### Refinement

All H atoms could have been discerned in a difference Fourier map. Nevertheless, all the H atoms attached to the carbon atoms were constrained in a riding motion approximation with C_aryl_—H = 0.93 and C_methyl_—H = 0.96 Å. The *U*_iso_ values were constrained to be 1.5*U*_eq_ of the carrier atom for methyl H atoms and 1.2*U*_eq_ for the remaining H atoms. A rotating group model was used for the methyl groups. The highest residual electron density peak is located at 1.01 Å from C6 and the deepest hole is located at 0.33 Å from S1.

### Crystal data

**Table:**

C_12_H_12_NS^+^·C_7_H_7_O_3_S^−^	*Z* = 2
*M**_r_* = 373.49	*F*(000) = 392
Triclinic, *P*1	*D*_x_ = 1.425 Mg m^−^^3^
Hall symbol: -P 1	Melting point = 507–509 K
*a* = 9.2947 (1) Å	Mo *K*α radiation, λ = 0.71073 Å
*b* = 9.6144 (1) Å	Cell parameters from 4606 reflections
*c* = 10.7790 (1) Å	θ = 2.4–29.0°
α = 87.817 (1)°	µ = 0.32 mm^−^^1^
β = 64.702 (1)°	*T* = 100 K
γ = 88.712 (1)°	Block, yellow
*V* = 870.21 (2) Å^3^	0.36 × 0.35 × 0.18 mm

### Data collection

**Table:**

Bruker SMART APEXII CCD area-detector diffractometer	4606 independent reflections
Radiation source: fine-focus sealed tube	4122 reflections with *I* > 2σ(*I*)
graphite	*R*_int_ = 0.023
Detector resolution: 8.33 pixels mm^-1^	θ_max_ = 29.0°, θ_min_ = 2.4°
ω scans	*h* = −12→12
Absorption correction: multi-scan (*SADABS*; Bruker, 2005)	*k* = −13→13
*T*_min_ = 0.893, *T*_max_ = 0.945	*l* = −14→14
18024 measured reflections	

### Refinement

**Table:**

Refinement on *F*^2^	Primary atom site location: structure-invariant direct methods
Least-squares matrix: full	Secondary atom site location: difference Fourier map
*R*[*F*^2^ > 2σ(*F*^2^)] = 0.051	Hydrogen site location: inferred from neighbouring sites
*wR*(*F*^2^) = 0.149	H-atom parameters constrained
*S* = 1.04	*w* = 1/[σ^2^(*F*_o_^2^) + (0.0809*P*)^2^ + 1.1333*P*] where *P* = (*F*_o_^2^ + 2*F*_c_^2^)/3
4606 reflections	(Δ/σ)_max_ = 0.001
228 parameters	Δρ_max_ = 0.98 e Å^−^^3^
0 restraints	Δρ_min_ = −0.71 e Å^−^^3^

### Special details

**Table:**

Experimental. The data was collected with the Oxford Cyrosystem Cobra low-temperature attachment.
Geometry. All e.s.d.'s (except the e.s.d. in the dihedral angle between two l.s. planes) are estimated using the full covariance matrix. The cell e.s.d.'s are taken into account individually in the estimation of e.s.d.'s in distances, angles and torsion angles; correlations between e.s.d.'s in cell parameters are only used when they are defined by crystal symmetry. An approximate (isotropic) treatment of cell e.s.d.'s is used for estimating e.s.d.'s involving l.s. planes.
Refinement. Refinement of *F*^2^ against ALL reflections. The weighted *R*-factor *wR* and goodness of fit *S* are based on *F*^2^, conventional *R*-factors *R* are based on *F*, with *F* set to zero for negative *F*^2^. The threshold expression of *F*^2^ > σ(*F*^2^) is used only for calculating *R*-factors(gt) *etc*. and is not relevant to the choice of reflections for refinement. *R*-factors based on *F*^2^ are statistically about twice as large as those based on *F*, and *R*- factors based on ALL data will be even larger.

### Fractional atomic coordinates and isotropic or equivalent isotropic displacement parameters (Å^2^)

**Table:**

	*x*	*y*	*z*	*U*_iso_*/*U*_eq_	
S1	0.15733 (7)	0.99833 (6)	0.16005 (6)	0.02891 (16)	
S2	0.55035 (5)	0.58728 (4)	0.20813 (4)	0.01428 (13)	
O1	0.69298 (18)	0.61236 (15)	0.08113 (15)	0.0220 (3)	
O2	0.40517 (17)	0.63620 (15)	0.19977 (15)	0.0194 (3)	
O3	0.56449 (18)	0.63482 (14)	0.32920 (14)	0.0196 (3)	
N1	−0.0092 (2)	0.50151 (17)	0.75333 (17)	0.0169 (3)	
C1	0.1573 (2)	0.6066 (2)	0.5395 (2)	0.0221 (4)	
H1A	0.2574	0.6145	0.4662	0.026*	
C2	0.1351 (2)	0.5142 (2)	0.6456 (2)	0.0208 (4)	
H2A	0.2199	0.4597	0.6437	0.025*	
C3	−0.1335 (2)	0.5809 (2)	0.7588 (2)	0.0195 (4)	
H3A	−0.2317	0.5721	0.8342	0.023*	
C4	−0.1159 (2)	0.6742 (2)	0.6539 (2)	0.0206 (4)	
H4A	−0.2025	0.7279	0.6586	0.025*	
C5	0.0314 (2)	0.6894 (2)	0.5397 (2)	0.0196 (4)	
C6	0.0632 (2)	0.7841 (2)	0.4220 (2)	0.0220 (4)	
H6A	0.1643	0.7827	0.3497	0.026*	
C7	−0.0453 (3)	0.8733 (2)	0.4121 (2)	0.0235 (4)	
H7A	−0.1461	0.8719	0.4849	0.028*	
C8	−0.0211 (3)	0.9713 (2)	0.2998 (2)	0.0225 (4)	
C9	−0.1401 (2)	1.0515 (2)	0.29383 (19)	0.0151 (3)	
H9A	−0.2454	1.0489	0.3591	0.018*	
C10	−0.0793 (3)	1.1440 (2)	0.1689 (2)	0.0247 (4)	
H10A	−0.1417	1.2086	0.1474	0.030*	
C11	0.0787 (3)	1.1230 (2)	0.0896 (2)	0.0262 (4)	
H11A	0.1367	1.1709	0.0070	0.031*	
C12	−0.0343 (3)	0.3981 (2)	0.8657 (2)	0.0241 (4)	
H12A	0.0649	0.3795	0.8710	0.036*	
H12B	−0.0740	0.3134	0.8479	0.036*	
H12C	−0.1099	0.4341	0.9510	0.036*	
C13	0.4960 (2)	0.1137 (2)	0.2619 (2)	0.0178 (4)	
C14	0.5220 (2)	0.1820 (2)	0.1374 (2)	0.0179 (4)	
H14A	0.5273	0.1303	0.0643	0.021*	
C15	0.5401 (2)	0.3257 (2)	0.12029 (19)	0.0161 (3)	
H15A	0.5573	0.3694	0.0367	0.019*	
C16	0.5323 (2)	0.40335 (18)	0.22945 (18)	0.0141 (3)	
C17	0.5046 (2)	0.33792 (19)	0.35510 (19)	0.0158 (3)	
H17A	0.4983	0.3899	0.4283	0.019*	
C18	0.4864 (2)	0.1940 (2)	0.3702 (2)	0.0174 (4)	
H18A	0.4674	0.1505	0.4543	0.021*	
C19	0.4833 (3)	−0.0423 (2)	0.2771 (3)	0.0260 (4)	
H19A	0.3967	−0.0675	0.3632	0.039*	
H19B	0.5807	−0.0809	0.2744	0.039*	
H19C	0.4644	−0.0779	0.2034	0.039*	

### Atomic displacement parameters (Å^2^)

**Table:**

	*U*^11^	*U*^22^	*U*^33^	*U*^12^	*U*^13^	*U*^23^
S1	0.0231 (3)	0.0296 (3)	0.0285 (3)	0.0008 (2)	−0.0063 (2)	0.0060 (2)
S2	0.0174 (2)	0.0119 (2)	0.0122 (2)	0.00110 (15)	−0.00523 (17)	0.00122 (15)
O1	0.0223 (7)	0.0188 (7)	0.0167 (7)	−0.0017 (5)	−0.0007 (6)	0.0020 (5)
O2	0.0224 (7)	0.0171 (6)	0.0200 (7)	0.0047 (5)	−0.0105 (6)	0.0004 (5)
O3	0.0284 (7)	0.0151 (6)	0.0183 (7)	0.0020 (5)	−0.0129 (6)	−0.0014 (5)
N1	0.0175 (7)	0.0161 (7)	0.0172 (7)	0.0001 (6)	−0.0076 (6)	−0.0002 (6)
C1	0.0187 (9)	0.0242 (10)	0.0180 (9)	−0.0016 (7)	−0.0028 (7)	−0.0009 (7)
C2	0.0163 (8)	0.0210 (9)	0.0229 (10)	0.0035 (7)	−0.0062 (7)	−0.0033 (7)
C3	0.0150 (8)	0.0234 (9)	0.0190 (9)	0.0000 (7)	−0.0061 (7)	−0.0007 (7)
C4	0.0182 (9)	0.0221 (9)	0.0235 (10)	0.0023 (7)	−0.0112 (8)	0.0002 (7)
C5	0.0252 (9)	0.0169 (9)	0.0181 (9)	−0.0040 (7)	−0.0104 (8)	0.0005 (7)
C6	0.0215 (9)	0.0222 (10)	0.0205 (9)	−0.0015 (7)	−0.0074 (8)	0.0003 (7)
C7	0.0218 (9)	0.0251 (10)	0.0218 (10)	−0.0018 (8)	−0.0077 (8)	0.0010 (8)
C8	0.0275 (10)	0.0195 (9)	0.0225 (10)	−0.0024 (8)	−0.0127 (8)	0.0015 (7)
C9	0.0091 (7)	0.0229 (9)	0.0135 (8)	0.0019 (6)	−0.0051 (6)	−0.0011 (7)
C10	0.0281 (10)	0.0218 (10)	0.0280 (11)	−0.0013 (8)	−0.0162 (9)	0.0061 (8)
C11	0.0295 (11)	0.0243 (10)	0.0242 (10)	−0.0038 (8)	−0.0115 (9)	0.0076 (8)
C12	0.0322 (11)	0.0195 (9)	0.0224 (10)	−0.0001 (8)	−0.0137 (9)	0.0034 (8)
C13	0.0158 (8)	0.0145 (8)	0.0243 (9)	0.0013 (6)	−0.0098 (7)	0.0004 (7)
C14	0.0181 (8)	0.0169 (9)	0.0187 (9)	0.0016 (7)	−0.0079 (7)	−0.0030 (7)
C15	0.0167 (8)	0.0172 (9)	0.0139 (8)	0.0014 (6)	−0.0061 (7)	0.0001 (6)
C16	0.0147 (8)	0.0123 (8)	0.0141 (8)	0.0012 (6)	−0.0051 (6)	0.0004 (6)
C17	0.0172 (8)	0.0160 (8)	0.0142 (8)	0.0013 (6)	−0.0069 (7)	0.0007 (6)
C18	0.0172 (8)	0.0165 (9)	0.0190 (9)	0.0000 (6)	−0.0086 (7)	0.0043 (7)
C19	0.0313 (11)	0.0141 (9)	0.0367 (12)	−0.0006 (8)	−0.0186 (10)	0.0020 (8)

### Geometric parameters (Å, °)

**Table:**

S1—C11	1.707 (2)	C9—C10	1.484 (3)
S1—C8	1.715 (2)	C9—H9A	0.9300
S2—O2	1.4569 (15)	C10—C11	1.362 (3)
S2—O3	1.4574 (14)	C10—H10A	0.9300
S2—O1	1.4587 (14)	C11—H11A	0.9300
S2—C16	1.7769 (18)	C12—H12A	0.9600
N1—C3	1.351 (2)	C12—H12B	0.9600
N1—C2	1.352 (3)	C12—H12C	0.9600
N1—C12	1.479 (3)	C13—C18	1.394 (3)
C1—C2	1.367 (3)	C13—C14	1.398 (3)
C1—C5	1.400 (3)	C13—C19	1.504 (3)
C1—H1A	0.9300	C14—C15	1.391 (3)
C2—H2A	0.9300	C14—H14A	0.9300
C3—C4	1.371 (3)	C15—C16	1.393 (3)
C3—H3A	0.9300	C15—H15A	0.9300
C4—C5	1.403 (3)	C16—C17	1.393 (3)
C4—H4A	0.9300	C17—C18	1.393 (3)
C5—C6	1.458 (3)	C17—H17A	0.9300
C6—C7	1.346 (3)	C18—H18A	0.9300
C6—H6A	0.9300	C19—H19A	0.9600
C7—C8	1.447 (3)	C19—H19B	0.9600
C7—H7A	0.9300	C19—H19C	0.9600
C8—C9	1.357 (3)		
			
C11—S1—C8	92.72 (11)	C11—C10—C9	112.07 (19)
O2—S2—O3	112.96 (8)	C11—C10—H10A	124.0
O2—S2—O1	113.06 (9)	C9—C10—H10A	124.0
O3—S2—O1	113.19 (9)	C10—C11—S1	111.84 (17)
O2—S2—C16	105.46 (9)	C10—C11—H11A	124.1
O3—S2—C16	105.73 (9)	S1—C11—H11A	124.1
O1—S2—C16	105.54 (8)	N1—C12—H12A	109.5
C3—N1—C2	120.63 (17)	N1—C12—H12B	109.5
C3—N1—C12	118.95 (17)	H12A—C12—H12B	109.5
C2—N1—C12	120.41 (17)	N1—C12—H12C	109.5
C2—C1—C5	120.85 (18)	H12A—C12—H12C	109.5
C2—C1—H1A	119.6	H12B—C12—H12C	109.5
C5—C1—H1A	119.6	C18—C13—C14	118.08 (18)
N1—C2—C1	120.52 (18)	C18—C13—C19	121.03 (18)
N1—C2—H2A	119.7	C14—C13—C19	120.87 (18)
C1—C2—H2A	119.7	C15—C14—C13	121.46 (18)
N1—C3—C4	120.49 (18)	C15—C14—H14A	119.3
N1—C3—H3A	119.8	C13—C14—H14A	119.3
C4—C3—H3A	119.8	C14—C15—C16	119.38 (17)
C3—C4—C5	120.62 (18)	C14—C15—H15A	120.3
C3—C4—H4A	119.7	C16—C15—H15A	120.3
C5—C4—H4A	119.7	C15—C16—C17	120.27 (17)
C1—C5—C4	116.87 (18)	C15—C16—S2	119.07 (14)
C1—C5—C6	117.82 (18)	C17—C16—S2	120.61 (14)
C4—C5—C6	125.30 (19)	C16—C17—C18	119.45 (17)
C7—C6—C5	123.75 (19)	C16—C17—H17A	120.3
C7—C6—H6A	118.1	C18—C17—H17A	120.3
C5—C6—H6A	118.1	C17—C18—C13	121.35 (18)
C6—C7—C8	126.6 (2)	C17—C18—H18A	119.3
C6—C7—H7A	116.7	C13—C18—H18A	119.3
C8—C7—H7A	116.7	C13—C19—H19A	109.5
C9—C8—C7	122.8 (2)	C13—C19—H19B	109.5
C9—C8—S1	112.58 (16)	H19A—C19—H19B	109.5
C7—C8—S1	124.57 (17)	C13—C19—H19C	109.5
C8—C9—C10	110.76 (17)	H19A—C19—H19C	109.5
C8—C9—H9A	124.6	H19B—C19—H19C	109.5
C10—C9—H9A	124.6		
			
C3—N1—C2—C1	−0.7 (3)	C8—C9—C10—C11	1.8 (3)
C12—N1—C2—C1	177.87 (19)	C9—C10—C11—S1	−0.7 (3)
C5—C1—C2—N1	−0.3 (3)	C8—S1—C11—C10	−0.32 (19)
C2—N1—C3—C4	1.0 (3)	C18—C13—C14—C15	0.9 (3)
C12—N1—C3—C4	−177.58 (19)	C19—C13—C14—C15	−177.44 (18)
N1—C3—C4—C5	−0.3 (3)	C13—C14—C15—C16	0.0 (3)
C2—C1—C5—C4	0.9 (3)	C14—C15—C16—C17	−0.8 (3)
C2—C1—C5—C6	−179.05 (19)	C14—C15—C16—S2	−178.34 (14)
C3—C4—C5—C1	−0.6 (3)	O2—S2—C16—C15	69.43 (16)
C3—C4—C5—C6	179.3 (2)	O3—S2—C16—C15	−170.67 (14)
C1—C5—C6—C7	−177.1 (2)	O1—S2—C16—C15	−50.48 (17)
C4—C5—C6—C7	3.0 (3)	O2—S2—C16—C17	−108.10 (16)
C5—C6—C7—C8	178.9 (2)	O3—S2—C16—C17	11.80 (18)
C6—C7—C8—C9	175.6 (2)	O1—S2—C16—C17	131.99 (16)
C6—C7—C8—S1	−3.7 (3)	C15—C16—C17—C18	0.7 (3)
C11—S1—C8—C9	1.40 (18)	S2—C16—C17—C18	178.16 (14)
C11—S1—C8—C7	−179.3 (2)	C16—C17—C18—C13	0.3 (3)
C7—C8—C9—C10	178.64 (19)	C14—C13—C18—C17	−1.0 (3)
S1—C8—C9—C10	−2.0 (2)	C19—C13—C18—C17	177.30 (18)

### Hydrogen-bond geometry (Å, °)

**Table:**

*D*—H···*A*	*D*—H	H···*A*	*D*···*A*	*D*—H···*A*
C2—H2A···O3^i^	0.93	2.31	3.219 (3)	166
C3—H3A···O1^ii^	0.93	2.49	3.168 (2)	130
C6—H6A···O2	0.93	2.56	3.378 (3)	147
C11—H11A···O1^iii^	0.93	2.54	3.303 (3)	139
C12—H12A···O1^i^	0.96	2.52	3.455 (3)	165
C12—H12C···O1^ii^	0.96	2.47	3.341 (3)	151
C15—H15A···O2^iv^	0.93	2.42	3.272 (2)	152
C17—H17A···O3^i^	0.93	2.43	3.202 (2)	141
C4—H4A···Cg1^v^	0.93	2.62	3.431 (2)	145
C10—H10A···Cg1^vi^	0.93	2.95	3.666 (3)	135

Symmetry codes: (i) −*x*+1, −*y*+1, −*z*+1; (ii) *x*−1, *y*, *z*+1;  (iii) −*x*+1, −*y*+2, −*z*; (iv) −*x*+1, −*y*+1, −*z*; (v) −*x*, −*y*+1, −*z*+1; (vi) *x*−1, *y*+1, *z*.
